# Lyophyllin, a Mushroom Protein from the Peptidase M35 Superfamily Is an RNA N-Glycosidase

**DOI:** 10.3390/ijms222111598

**Published:** 2021-10-27

**Authors:** Jia-Qi Lu, Wei-Wei Shi, Meng-Jie Xiao, Yun-Sang Tang, Yong-Tang Zheng, Pang-Chui Shaw

**Affiliations:** 1 Centre for Protein Science and Crystallography, School of Life Sciences, The Chinese University of Hong Kong, Hong Kong, China; lujq@link.cuhk.edu.hk (J.-Q.L.); mengjiexiao@link.cuhk.edu.hk (M.-J.X.); samtys0910@gmail.com (Y.-S.T.); 2 Li Dak Sum Yip Yio Chin R & D Centre for Chinese Medicine, The Chinese University of Hong Kong, Hong Kong, China; 3 BayRay Innovation Center, Shenzhen Bay Laboratory, Shenzhen 518107, China; shiww@szbl.ac.cn; 4 Key Laboratory of Animal Models and Human Disease Mechanisms, National Kunming High Level Biosafety Research Center for Non-Human Primates, Kunming Institute of Zoology, Chinese Academy of Sciences, Kunming 650223, China; zhengyt@mail.kiz.ac.cn

**Keywords:** ribosome-inactivating proteins, lyophyllin, peptidase M35 superfamily, N-glycosidase

## Abstract

Ribosome-inactivating proteins (RIPs) hydrolyze the N-glycosidic bond and depurinate a specific adenine residue (A-4324 in rat 28S ribosomal RNA, rRNA) in the conserved α-sarcin/ricin loop (α-SRL) of rRNA. In this study, we have purified and characterized lyophyllin, an unconventional RIP from *Lyophyllum shimeji*, an edible mushroom. The protein resembles peptidase M35 domain of peptidyl-Lys metalloendopeptidases. Nevertheless, protein either from the mushroom or in recombinant form possessed N-glycosidase and protein synthesis inhibitory activities. A homology model of lyophyllin was constructed. It was found that the zinc binding pocket of this protein resembles the catalytic cleft of a classical RIP, with key amino acids that interact with the adenine substrate in the appropriate positions. Mutational studies showed that E122 may play a role in stabilizing the positively charged oxocarbenium ion and H121 for protonating N-3 of adenine. The tyrosine residues Y137 and Y104 may be used for stacking the target adenine ring. This work first shows a protein in the peptidase M35 superfamily based on conserved domain search possessing N-glycosidase activity.

## 1. Introduction

Ribosome inactivating proteins (RIPs) are a group of RNA N-glycosidases that catalyze the depurination of A-4324 in the conserved α-sarcin/ricin loop (α-SRL) of the 28S ribosomal RNA [1], and thus inhibit protein synthesis, resulting in cell death. RIPs possess various biological activities, including anti-tumor [2], anti-HIV, and anti-plant viruses [3]. Besides, RIPs also show DNase, RNase, phospholipase, chitinase, superoxide dismutase (SOD), and apoptosis inducing activities [4,5]. These activities were assayed by various methods. For example, cell-free protein synthesis inhibitory activity was used to assess the ribosome-inactivating activity [6,7,8,9]. Depurination assay based on the release of β-fragment from ribosomal RNA after aniline treatment was used to assess the N-glycosidase activity [10,11,12,13]. MTT assay was used to measure the cytotoxicity and anti-tumor activity [14,15,16,17].

RIPs can be divided into three types according to their protein domain composition. Type I RIPs such as trichosanthin (TCS) from *Trichosanthes kirilowii* are composed of a single polypeptide chain about 30 kDa. Type II RIPs, such as ricin from *Ricinus communis*, consist of a toxic A chain and a lectin binding domain linked with a disulphide bond [18,19]. Type III RIPs such as maize ribosome-inactivating protein needs the removal of an internal segment to activate its N-glycosidase activity [20].

The active site of an RIP has several conserved residues that play important roles in the mechanism. For example, trichosanthin (TCS) has five conserved catalytic residues, Tyr70, Tyr111, Glu160, Arg163, and Phe192 [17]. RIPs share a similar ‘RIP fold’ in their overall three-dimensional structure with the catalytic residues located at the active cleft between the N-terminal and C-terminal domain [21].

RIPs are mostly found in plants and some have also been found in bacteria, fungi, and possibly other eukaryotic organisms [22,23]. Several RIPs have been found in mushrooms; including volvarin from *Volvariella volvacea* [24] and Flammulin from *Flammulina velutipes* [25]. RIPs from mushroom have unique molecular masses and their N-terminal sequences are distinct from classical RIPs, suggesting a novel mechanism to achieve the RIP activities.

Enzymes usually contain one active site and catalyze a single enzymatic reaction. However, some enzymes use the same active site or different active sites for different catalytic reactions. Examples of the former include fructose-1,6-bisphosphate aldolase/phosphatase [26] and siroheme synthase with dehydrogenase and chelatase activities [27]. Examples of the latter include Kemp eliminase with dual regioselectivity in galactan polymerization and glucosamine-6-phosphate synthase catalyzing the first and rate-limiting step in hexosamine metabolism [28]. In some circumstances, an enzyme may have a site resembling the active site of an unrelated enzyme and carries out the catalytic reaction of the unrelated enzyme. An example is the functional diversity of the TIM barrel, in which the superfamily shares active-site residues invariably at C termini of β strands with more than 60 different enzymatic functions [29].

Lyophyllin from *Lyophyllum shimeji*, which has molecular weight of 20 kDa, has been reported with ribosome-inactivating and HIV-1 reverse transcriptase inhibitory activities [30]. It also exerts deleterious effects on mouse embryonic development [31]. Its N-terminal sequence was found to be different from the classical RIPs. Nevertheless, there is no information on the nature of this enzyme and the mechanism of action. We set forth to characterize lyophyllin and suggest that this protein belongs to the peptidase M35 superfamily and it can act as an RNA N-glycosidase.

## 2. Results

### 2.1. Purification and Identification of Lyophyllin

**Figure 1:**
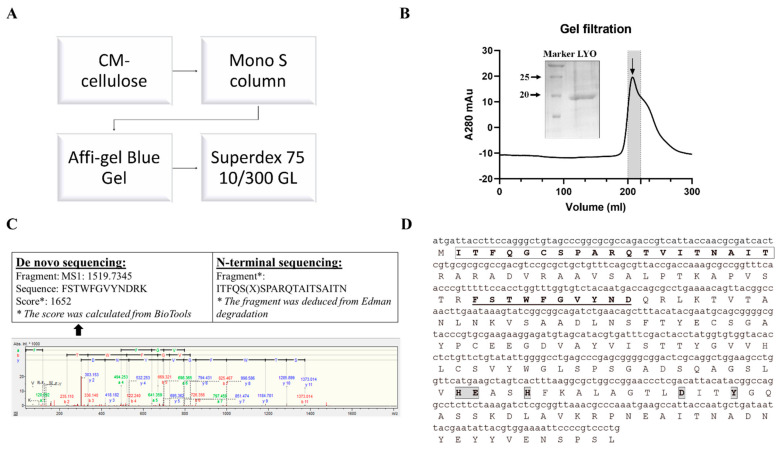
Purification and characterization of lyophyllin from *Lyophyllum shimeji*. Fresh mushroom fruiting body of *L. shimeji* was grounded in saline buffer and purified as stated in Section 4. (**A**) Flowchart of purification steps of lyophyllin from *Lyophyllum shimeji*. (**B**) Gel filtration profile of the final purification step of lyophyllin and related 15% SDS-PAGE gel photo. LYO: lyophyllin (**C**) The N-terminal sequence obtained by Edman digestion and de novo sequencing analysis result determined by MALDI-TOF-MS. * De novo sequencing score was calculated from BioTools as stated in Section 4. (**D**) Identified DNA and protein sequence of lyophyllin according to its N-terminal sequencing and de novo sequencing data. The sequence that matches the N-terminal sequencing result is framed and in bold. The sequences that match the MALDI-TOF-MS data are underlined and in bold. The framed and highlighted amino acids are the “HEXXH+D+Y” active motif of the M35 metalloendopeptidase superfamily.

N-glycosidase activity guided purification was applied to purify lyophyllin. Lyophylllin from *L. shimeji* was purified after passing the extract through three columns (Figure 1A,B) according to previous study [30]. The yield was 0.5 mg from 1 kg of fresh *L. shimeji* fruiting body. The purified protein was then sequenced by MALDI-TOF-MS and N-terminal sequencing (Figure 1C). The DNA and protein sequence of lyophyllin were found (Figure 1D) by comparing the peptides with the genome of *L. shimeji* (GenBank: BCJS00000000.1).

### 2.2. Heterogeneous Expression and Ribosome-Inactivating Activity of Recombinant Lyophyllin

**Figure 2:**
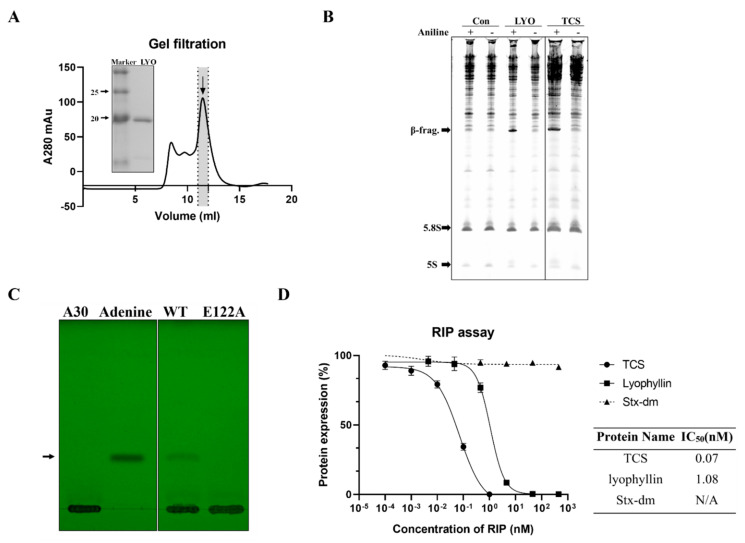
N-glycosidase activity and protein inhibitory activity of recombinant lyophyllin. (**A**) SDS-PAGE gel and gel filtration profile of recombinant lyophyllin. (**B**) N-glycosidase activity of lyophyllin on *Saccharomyces cerevisiae* yeast 80S ribosome. TCS: trichosanthin. Aniline: with or without aniline treatment after reaction as stated in Section 4**.** β-frag.: β-fragment released from RIPs after aniline treatment. (**C**) A30-ssDNA was incubated with respective protein at 37 °C for 240 min and then separated by TLC. The arrow indicates the released adenine. A30: A30-ssDNA; Adenine: adenine only group, worked as control. WT: wildtype lyophyllin protein; E122A: lyophyllin E122 to alanine variant. (**D**) The protein synthesis inhibitory activity of lyophyllin based on cell-free protein synthesis system, as stated in Section 4, was compared with TCS and Shiga toxin E167K/R176K double mutant. The calculated IC_50_ of the protein inhibitory activities by GraphPad Prism are listed. n ≥ 3.

Recombinant lyophyllin was purified (Figure 2A) and it exhibited N-glycosidase activity (Figure 2B). It also depurinated and cut the N-glycosidic bond of an A30-ssDNA, mimicking the α-sarcin/ricin loop [32], and released adenine (Figure 2C). This activity was lost after mutating E122 to alanine. The activity of lyophyllin to inhibit protein synthesis was also measured on a cell free system. Inhibitory activity was dose dependent with IC_50_ of 1.08 nM (Figure 2D).

### 2.3. Sequence Analysis and In Silico Homology Modeling of Lyophyllin

**Figure 3:**
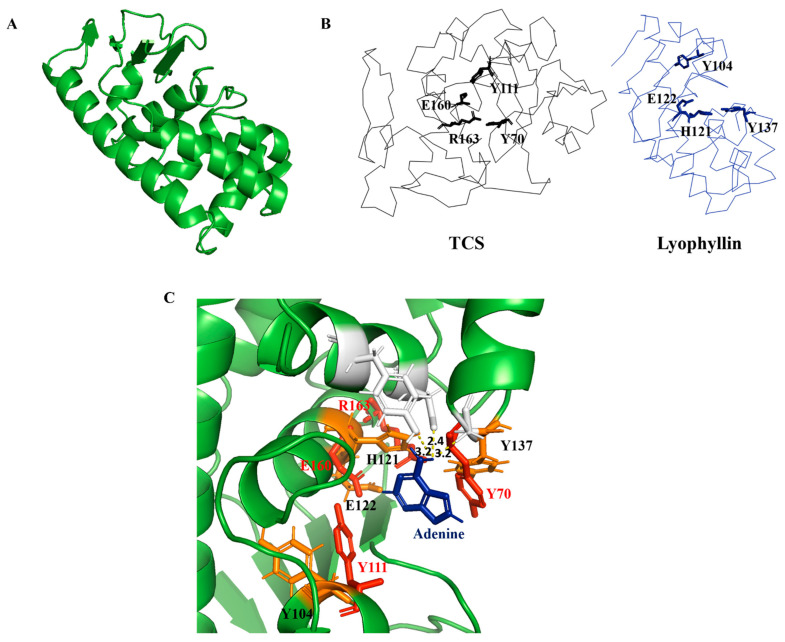
Homology modelling and sequence analysis of lyophyllin. (**A**) Homology modeled structure of lyophyllin by RoseTTAFold (https://robetta.bakerlab.org/, accessed date: 22 July 2021). (**B**) Structures of trichosanthin (TCS, PDB code: 1TCS) and lyophyllin were shown in black and blue ribbon. The active site residues of TCS and the corresponding residues of lyophyllin were shown in sticks. (**C**) Docking of adenine molecule to the lyophyllin structure with the superposition of the active site residues of TCS (in red color) and the corresponding residues of lyophyllin (in orange color).

Conserved domain (CD) search [33] showed lyophyllin resembles peptidase M35 domain of peptidyl-Lys metalloendopeptidases (MEP, EC 3.4.24.20) (Figure S1A). Proteins in this superfamily specifically cleave -X-Lys- bonds (where X may even be Pro) in proteins and peptides. However, this activity was not found in lyophyllin with or without Zn^2+^ and EDTA (Figure S3). An in-silico homology modeling of lyophyllin was made by the improved deep learning-based method, RoseTTAFold [34] (Figure 3A). The resulting structure echoed secondary structure analysis of lyophyllin by circular dichroism (CD) spectroscopy that both structures contained mainly helixes and coils, but only minimal percentage of strands (Figure S2).

It was found that several amino acids in the zinc binding motif of lyophyllin have orientation similar to the conserved amino acids in TCS active site cleft (Figure 3B). According to the superposition of hypothetic active site of lyophyllin and that of TCS, the E122, H121, Y137, and Y104 may be equivalent to E160, R163, Y70, and Y111 of TCS. Docking of adenine to the active pocket of this model showed hydrogen bonds between adenine and Y162, D158, and Y137 (Figure 3C). The adenine ring is inserted between Y104 and Y137, with a distance of 3.2 Å from Y137. GAGA of the α-sarcin/ricin loop was also docked (Figure S4). The first adenosine in GAGA, which corresponds to A-4324 was able to be aligned with the single adenine with a little clash on Y137. Such a clash could probably be avoided if there was a conformational change upon the rotation of Y137 while stacking to A-4324 in GAGA.

### 2.4. Revealing the Mechanism of Action of Lyophyllin by Mutational Studies

**Figure 4:**
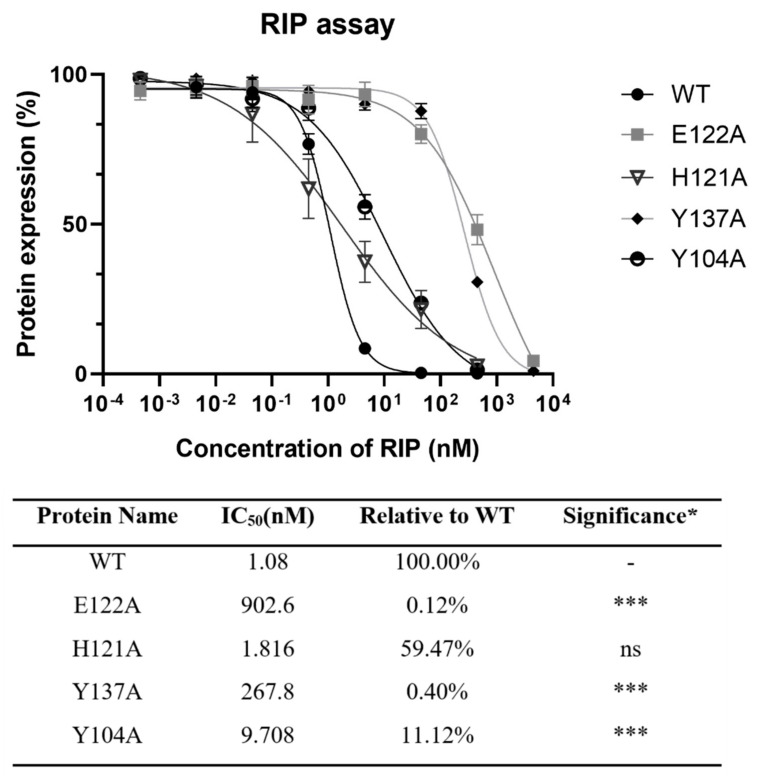
Protein synthesis inhibitory activity assay based on cell-free protein synthesis system as stated in Section 4 was conducted on lyophyllin and its variants. IC_50_ of lyophyllin and its variants on inhibiting protein synthesis are listed. n ≥ 3. * Significance was analyzed by Graphpad Prism using one-way ANOVA, Dunnett’s multiple comparisons test (compared with WT group). Significance was shown by *p* value. *** *p* < 0.001. ns: not statistically significant. WT: wild type.

**Figure 5:**
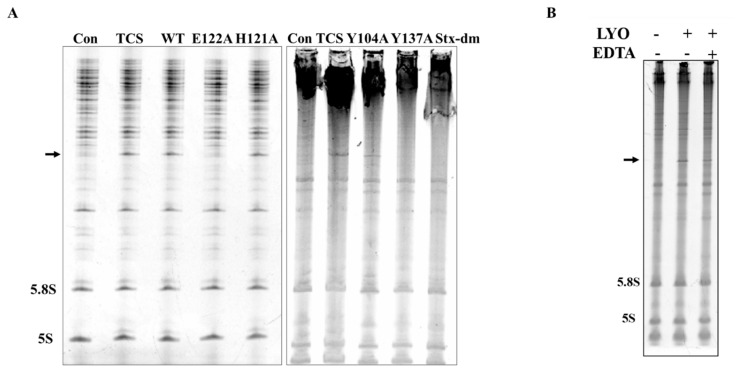
N-glycosidase activity of lyophyllin and its variants. *Saccharomyces cerevisiae* yeast 80S ribosome were used to test depurination activity assay as described in Section 4. (**A**) The N-glycosidase activity of wildtype lyophyllin and the indicated variants of lyophyllin. The arrow indicates the β-fragment suggesting N-glycosidase activity. (**B**) The N-glycosidase activity of lyophyllin with or without EDTA. LYO: lyophyllin; EDTA: EDTA final concentration at 25 mM in the reaction mix.

To find the importance of E122, H121, and Y137 in the zinc binding motif and Y104 nearby for the activity of lyophyllin, these amino acids were mutated to alanine. Protein synthesis inhibitory activity assay (also called RIP assay in this paper) showed variants Y137A and E122A had a drastically decrease in RIP activities by 248- and 836-fold respectively, showing these two amino acids are important for the RIP activity of lyophylin (Figure 4). Depurination assay also showed similar trends (Figure 5A).

### 2.5. Cytotoxicity of Mushroom RIP Lyophyllin on Cancer Cell Line Hela, HepG2, and JAR

**Figure 6:**
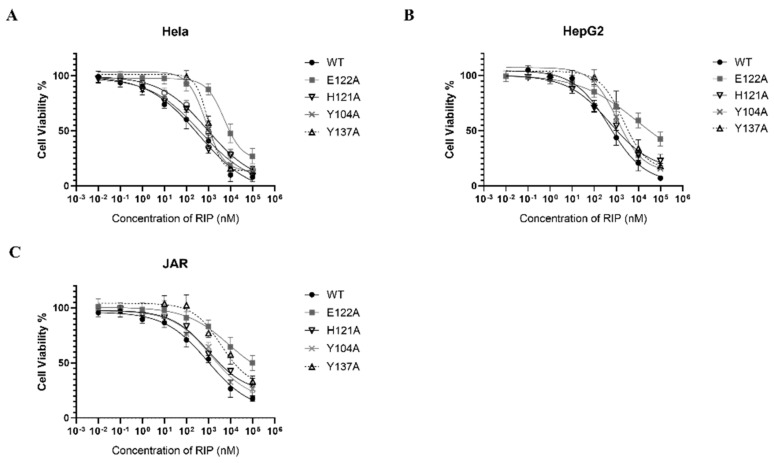
Cytotoxicity of lyophyllin and its variants on cancer cell lines (**A**) Hela, (**B**) HepG2, (**C**) JAR. Cells were seeded in 96-well plates and treated with increasing amounts of wildtype lyophyllin or its variants for 48 h as stated in Section 4. Values are shown by the mean ± standard deviation of over three independent experiments.

RIPs are toxic to a broad range of tumor cells in vitro and in vivo [4,35]. Lyophyllin was also found exhibiting dose dependent cytotoxicity on cancer cell lines Hela, HepG2, and JAR, with IC_50_ 358.8, 489.8, and 926.9 nM respectively (Figure 6). The cytotoxicity of E122A and Y137A variants to Hela, HepG2, and JAR cells had significantly reduced (Table 1), suggesting its cytotoxicity on cancer cells was correlated to the RIP activity.

## 3. Discussion

Classical RIPs are RNA N-glycosidases which can specifically recognize and remove an adenine on the α-sarcin/ricin loop on 28S/23S rRNA, resulting in the inhibition of protein synthesis [36]. Over the years, a number of RIPs with distinctive molecular weights and N-terminal protein sequences have been discovered [25,30,37,38,39,40,41]. Among them, lyophyllin in *L. shimeji*, a common edible mushroom, is of special interest. Lyophyllin depurinates ribosomal RNA and inhibits protein synthesis [30]. It also inhibits fungal growth and HIV-1 reverse transcriptase activity, and hampers mouse embryonic development [31].

In this study, we set forth to reveal the identity of lyophyllin and characterize its ribosome-inactivating mechanism. We first elucidated the DNA and protein sequences of lyophyllin and found that it belongs to the peptidase M35 like superfamily, which includes a “HEXXH+D+Y” active pocket (Figure S1A). However, it did not show peptidase activity on BSA and TCS after incubating 48 h at 25 °C (Figure S3) and 37 °C (data not shown) with or without EDTA and zinc. A similar example has also been shown in LECT2, an M23 metalloendopeptidase, which possesses conserved motifs (HXXXH and HXH) but lacks protease activity [42]. This is due to the blocked zinc binding groove by a protruding structural element in the vicinity. By structural alignment of lyophyllin and GfMEP (PDB code: 1g12), a member of M35 endopeptidase superfamily, lyophyllin is found to have an additional intrachain loop (amino acid 83–87) which may block the peptidase activity (Figure S1B). Alignment results between lyophyllin and other functional M35 like peptidases indeed showed that the latter did not have this additional intrachain loop (Figure S1A).

Recombinant lyophyllin shows a dose dependent protein synthesis inhibitory activity with IC_50_ of 1.08 nM. It is close to the reported value of 1 nM [30]. After removing the zinc ion by EDTA, lyophyllin still elicits the N-glycosidase activity (Figure 5B), showing that zinc binding is not required for the activity. In classical RIPs, five highly conserved amino acids are found to be crucial to the RNA N-glycosidase activity. For example, in TCS, the invariant catalytic residues include Y70, Y111, R163, E160, and W192. The adenine base stacks between the two aromatic side chains of Y70 and Y111. The side chains of R163 and E160 can form hydrogen bonds to the N(3) of the adenine and O(3′) of adenosine, respectively [43]. In the catalytic process, the adenine ring is inserted between the aromatic rings of Y70 and Y111, the N-glycosidase bond of the adenine is broken by partial protonation of R163, while E160 serves as a general base to polarize the attacking water molecule [44,45,46].

Alignment of lyophyllin with classical RIPs—including trichosanthin, ricin A chain, and shiga toxin—were conducted. Lyophyllin has low sequence similarity to the classical RIPs. We have generated a structural model of lyophyllin and it is not possible to align this model to the classical RIPs (Figure 3B). However, the region on lyophyllin for zinc binding can form a pocket for binding adenine. The orientation of amino acid Y104 and the zinc binding residues E122, H121, and Y137 of lyophyllin in three dimensions highly resembles E160, R163, Y70, and Y111 of TCS (Figure 3C).

To find the importance of these residues, several variants of lyophyllin were generated by site-direct mutagenesis. The depurination activity of E122A was drastically disrupted, and its RIP activity decreased by 836-fold. The RIP activities of Y137A and Y104A decreased for about 245- and 9-fold respectively, showing the greater importance of the Y137.

**Figure 7:**
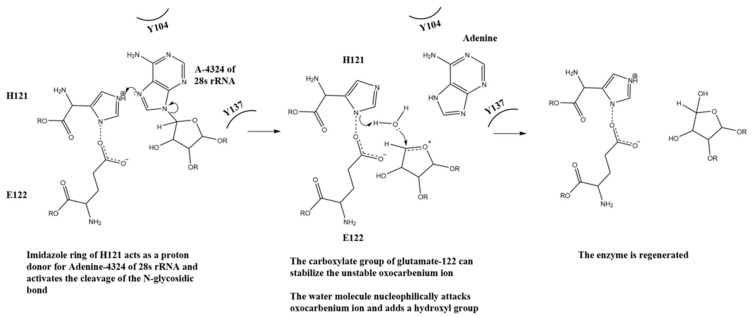
A putative mechanism for the N-glycosidase activity of lyophyllin.

With the above observation, we propose a mechanism for the N-glycosidase activity of lyophyllin (Figure 7). H121 takes the role of R163 in TCS for protonating and breaking the N-glycosidase bond, and E122 takes the role of E160 for stabilizing the positively charged oxocarbenium ion and polarize the attacking water molecule. Y137 and Y104 take the role of Y70 and Y111 in TCS for stacking the target adenine ring. The relative activities of these residues in lyophyllin are corelated to those in ricin. For the latter, mutation of Y80 to Phe results in a 15-fold loss of RIP activity to wild type, while mutation Y123 to Phe results in 7-fold reduction [47].

Why does lyophyllin possess RIP activity? In plants, RIPs have been found to enhance defense response against pathogens and insect pest attacks [48,49]. Lyophyllin may therefore give an additional advantage of pathogen protection to the mushroom. Since lyophyllin does not possess a peptidase activity, it would be of interest to find if other functional proteins in the M35 superfamily also possess RIP activities.

## 4. Materials and Methods

### 4.1. Purification, Characterization, and Identification of Lyophyllin

Fresh fruiting bodies of *Lyophyllum shimeji* from local market were ground in liquid nitrogen along with acid-washed sands for maximum cell disruption. The powder was then dissolved in extraction buffer (Normal saline 0.9 g NaCl/100 mL ddH_2_O) and centrifuged for 30 min at 11,655× *g* (Rotor JA-14, Beckman Coulter, Indianapolis, IN, USA) to remove fibrous materials. Ammonium sulfate was added gradually to the supernatant (20–80% saturation) with continuous stirring at 4 °C. After centrifugation, the pellet was dissolved in 20 mM Tris, pH 7.5, 100 mM NaCl, followed by exhaustive dialysis against the same buffer. The dialysate was centrifuged at 31,360× *g* (Rotor JA-20, Beckman Coulter, Indianapolis, IN, USA) for 60 min and passed through a 0.45 μm syringe filter to remove precipitates. The protein solution was concentrated to proper volume.

For further purification, ion exchange chromatography on CM-cellulose/Mono S and Affi-gel Blue Gel were used according to previous study [30]. Briefly, proteins in 10 mM ammonium acetate (pH 4.6) were loaded on CM-cellulose (Cytiva, Marlborough, MA, USA) and fraction containing lyophyllin was eluted by 50 mM ammonium acetate (pH 7.0). Buffer was then changed to 10 mM Tris (pH 7.2) and loaded on Affi-gel Blue Gel (Bio-Rad). Eluted fraction was then applied to Mono S column (Cytiva, Marlborough, MA, USA) in 10 mM ammonium acetate (pH 4.6) and then eluted with linear concentration gradient (0–1 M). The purification steps were guided by relative RIP activity test. FPLC with Superdex 75 10/300 GL (Cytiva, Marlborough, MA, USA) was used for the final step purification.

### 4.2. RNA N-Glycosidase Activity Assay

Yeast ribosomes from *Saccharomyces cerevisiae* were isolated and purified according to the published method [10]. The final concentration of ribosome was determined by A260 as described previously [50]. The reaction was carried out as follows: 4 μL fraction sample/purified protein (about 10 μg) and 6 μL 10X RIP buffer (2.5 M Tris buffer, pH 7.6, 250 mM KCl and 50 mM MgCl_2_) were added to 50 µL 2 A260 absorbance unit ribosomes. The reaction mix was then incubated at 37 °C for 30 min and terminated by adding of 0.1% SDS. Total RNA was extracted with a Trizol kit (Promega, Madison, WI, USA) and then treated with 1 M aniline and 0.8 M acetic acid working solution for 30 min on ice. For the control group without aniline treatment, this step was skipped. After incubation at 65 °C for 10 min, the RNA was separated with 8 M Urea/6% acrylamide Urea-gel. The released β-fragment was visualized with ethidium bromide. As an indication of β-fragment, TCS was used as a positive control.

### 4.3. Cell-Free Protein Synthesis Inhibitory Activity by RIP Assay

Luciferase mRNA (Promega, Madison, WI, USA) was introduced into the rabbit reticulocyte lysate translation system (Promega, Madison, WI, USA) as the protein synthesis reporter. The scale-down protein synthesis reaction was carried out as follows: 7 µL rabbit reticulocyte lysate, 0.1 µL of 1 mM amino acid mixture, 2 µL of RNasin Ribonuclease Inhibitor (40 U/µL, Promega, Madison, WI, USA), 0.4 µL of luciferase control RNA (1 μg/μL, Promega, Madison, WI, USA), 1 μL diluted RIP to different concentration, nuclease-free water to the final volume of 10 µL. The translation reaction mixture was incubated at 30 °C for 90 min. Then, 2.5 µL reaction mixture was added to 50 µL one-glo luciferase substrate (Promega, Madison, WI, USA). Luminescence was measured by the luminometer (CLARIOstar, BMG Labtech, Ortenberg, Germany). IC_50_ was calculated by Graphpad Prism 8.3.0 using a nonlinear regression model (four parameters).

### 4.4. Determination of DNA and Protein Sequence of Lyophyllin

Ultraflextreme MALDI-TOF-MS System (Bruker, Billerica, MA, USA) was used for peptide mass analysis. SDS-PAGE gel band was cut and pulverized by a clean needle, followed by destaining with 200 μL 50% MeOH/10 mM NH_4_HCO_3_ several times and dehydrating with acetonitrile (ACN). The product was then digested in 20 ng/μL trypsin digestion for 4 °C overnight. Then the peptides were extracted by 10 min sonication in 5 μL 80% acetonitrile/2.5% TFA and 1.5 μL peptide solution was spotted onto MALDI target plate and then covered by 0.5 μL matrix (Sigma-Aldrich, St Louis, MO, USA). The peptides with *m/z* around 550 to 4000 were detected using MALDI-TOF/TOF mass spectrometer. Standard porcine trypsin autolytic products (*m*/*z* 842.509, 1045.564, 1940.935, and 2211.104) was used to conduct the internal calibration. Contaminants, including trypsin autolytic products were excluded with a mass tolerance of ±0.2 Da. Spectra was interpreted by software assisted de novo sequence analysis (BioTools, Bruker Daltonics GmbH, Bremen, Germany). For N-terminal sequencing, PVDF membrane was sent to Applied Protein Technology (APTBIO, Shanghai, China) to analyze its N-terminal sequence based on Edman degradation using PPSQ-33A peptide sequencer (Shimadzu, Kyoto, Japan).

Using N-terminal sequence and de novo sequencing fragments as seeds, sequence of lyophyllin was identified in the genome of *Lyophyllum shimeji* (GenBank: BCJS00000000.1). Conserved domain search was conducted in the conserved domain database in 2020 [33] with default parameters.

### 4.5. Cloning, Heterologous Expression, and Purification of Lyophyllin and Variants

The DNA sequence of lyophyllin was synthesized by Integrated DNA technologies, IDT. The sequence was cloned to pET-28a vector with 6X His tag. Constructed plasmid was transformed to OverExpress C43(DE3) cell (Cat. No. CMC0019, Sigma, St Louis, MO, USA) and grown until OD600 0.8, then induced by 0.1 mM IPTG at 25 °C overnight. The yield was about 50 μg lyophyllin/L cells. Corresponding variants were made by site-direct mutagenesis (Table S1).

To express the recombinant protein, *E. coli* cells were harvested by centrifugation at 11,655× *g* (Rotor JA-14, Beckman Coulter, Indianapolis, IN, USA) for 4 min, and then lysed by flow cell disrupter JN-Mini (JNBIO, Guangdong, China) at 1200 bar and 4 °C in buffer A (20 mM Tris pH 7.5, 100 mM NaCl, 50 mM Imidazole, 5% glycerol). The nickel NTA beads (QIAGEN, Venlo, The Netherlands) were equilibrated in the same buffer before loaded with the cell lysate. Beads were washed by buffer A for 10X column volume (CV) and then buffer B (20 mM Tris pH 7.5, 100 mM NaCl, 100 mM Imidazole, 5% glycerol) for another 10 CV. Finally, protein was eluted by buffer C (20 mM Tris pH 7.5, 100 mM NaCl, 300 mM Imidazole, 5% glycerol). It was then concentrated to suitable volume and injected to AKTA Prime (Cytiva, Marlborough, MA, USA) with Superdex 75 10/300 GL gel filtration column (Cytiva, Marlborough, MA, USA).

### 4.6. Adenine-Releasing Assay by Thin Layer Chromatography

Adenine-releasing assay was performed as previous reported [32] with minor modification. In the 10 μL reaction mixture, 30 μg RIP was incubated with 20 μg A30-ssDNA (5′-AAAAAAAAAAAAAAAAAAAAAAAAAAAAAAAA-3′) and then separated on TLC silica gel F254 plates (Merck, Darmstadt, Germany) with eluent consisting of a mixture of acetonitrile/water/ammonia (32%) before 10 min saturation. Volume ratio was 18:1.6:0.6. After air drying, the spots were visualized by UV Lamp at 254 nm. 1 ug Adenine (Sigma, St Louis, MO, USA) dissolved by the same buffer was used as a control.

### 4.7. Peptidyl-Lys Metalloendopeptidase Assay

BSA (Bovine Serum Albumin, Sigma-Aldrich, St Louis, MO, USA) was used as a substrate for the peptidyl-Lys metalloendopeptidase assay as previously reported [51]. 10 µg of lyophyllin was used for each digestion experiment. BSA was first incubated with 50 mM dithiothreitol (DTT) at 50 °C for 15 min, and then followed by 100 mM Iodoacetamide at room temperature for 15 min. The protein sample was treated with lyophyllin at a ratio of 1/50 (w/w) at 25 °C (or 37 °C) for corresponding time. The final volume was adjusted to 200 μL. The reaction mix was stopped by loading dye and analyzed by 15% SDS-PAGE.

### 4.8. Homology Modeling of Lyophyllin and Docking of Adenine to the Putative Active Site

Homology model of lyophyllin was generated by improved deep learning based modelling method RoseTTAFold (https://robetta.bakerlab.org/, accessed date: 22 July 2021) [34]. The secondary structure from circular dichroism (CD) spectrum was analyzed by CAPITO (https://data.nmr.uni-jena.de/capito/index.php, accessed date: 28 April 2021) [52]. Docking of adenine to lyophyllin was conducted by SwissDock (http://www.swissdock.ch/, accessed date: 31 August 2021) [53].

### 4.9. Anti-Tumor Activity of Lyophyllin

Cell lines HeLa, HepG2 and JAR were used to test the cytotoxicity of lyophyllin based on 3-(4,5-dimethylthiazol-2-yl)-2,5-diphenyltetrazolium bromide (MTT) assay [54]. Cells were seeded in a 96-well plate at the density of 1 × 10^4^ cell/well in DMEM (Gibco, Thermo Fisher Scientific, Waltham, MA, USA) without 10% FBS (RPMI 1640 Medium for JAR), The cells were starved overnight to eliminate the effect of serum. Cells were then treated with lyophyllin or other RIPs at appropriate concentration. Lyophyllin and its variants were firstly buffer exchanged to 1X PBS (Gibco, Thermo Fisher Scientific, Waltham, MA, USA) and then diluted to appropriate concentration with the same cell culture medium. After incubation at 37 °C in 5% CO_2_ for 48 h, 10 μL MTT (5 mg/mL) was added to each well and then incubated in the same condition for 4 h. Then the culture medium was removed, and dimethyl sulfoxide (DMSO) (100 μL/well) was added to dissolve the formazan crystal. The absorbance of each well was measured at 570 nm by CLARIOstar multi-mode microplate reader (BMG Labtech, Ortenberg, Germany). The IC_50_ (50% inhibition of cell growth) was calculated by Graphpad Prism.

## Figures and Tables

**Table 1:** Cytotoxicity of lyophyllin and its variants on cancer cell lines. The IC_50_ of wildtype lyophyllin and its variants on cancer cells are listed. n ≥ 3. * Significance was analyzed by Graphpad Prism using one-way ANOVA, Dunnett’s multiple comparisons test (compared with WT group). Significance was shown by *p* value. *** *p* < 0.001. WT: wild type.

Protein Name	Hela			HepG2			JAR		
	IC_50_ (nM)	Relative to WT	Significance *	IC_50_ (nM)	Relative to WT	Significance *	IC_50_ (nM)	Relative to WT	Significance *
**WT**	358.8	100.00%	-	489.8	100.00%	-	926.9	100.00%	-
**E122A**	5148.0	6.97%	***	9039.0	5.42%	***	8557.0	10.83%	***
**H121A**	429.0	83.64%	***	525.9	93.14%	***	1009.0	91.86%	***
**Y104A**	760.6	47.07%	***	1104.0	44.36%	***	1284.0	72.18%	***
**Y137A**	1003.0	35.77%	***	2078.0	23.57%	***	3671.0	25.25%	***

## Data Availability

All data are contained within this article. The protein sequence data reported in this paper will appear in the UniProt Knowledgebase under the accession number C0HLZ4.

## Supplementary Materials

The following are available online at https://www.mdpi.com/article/10.3390/ijms222111598/s1.
