# Supplementary material for: Lyophyllin, a Mushroom Protein from the Peptidase M35 Superfamily Is an RNA N-Glycosidase
Source: Int J Mol Sci. 2021 Oct 27;22(21):11598. doi: 10.3390/ijms222111598 (PMC8584072; doi:10.3390/ijms222111598)
Supplement: Supplementary file 1 [file ijms-22-11598-s001.zip › ijms-1426924-supplementary.pdf]

Supplementary materials

Lyophyllin, a mushroom protein from the peptidase M35 superfamily Is an RNA N-glycosidase

Jia-Qi Lu [1,2], Wei-Wei Shi [3], Meng-Jie Xiao [1,2], Yun-Sang Tang [1,2], Yong-Tang Zheng [4] and Pang-Chui Shaw [1,2,*]

[1] Centre for Protein Science and Crystallography, School of Life Sciences, The Chinese University of Hong Kong, Hong Kong, China; lujq@link.cuhk.edu.hk (J.-Q.L.); mengjiexiao@link.cuhk.edu.hk (M.-J.X.); samtys0910@gmail.com (Y.-S.T.)

[2] Li Dak Sum Yip Yio Chin R & D Centre for Chinese Medicine, The Chinese University of Hong Kong, Hong Kong, China

[3] BayRay Innovation Center, Shenzhen Bay Laboratory, Shenzhen 518107, China; shiww@szbl.ac.cn

[4] Key Laboratory of Animal Models and Human Disease Mechanisms, National Kunming High Level Biosafety Research Center for Non-Human Primates, Kunming Institute of Zoology, Chinese Academy of Sciences, Kunming 650223, China; zhengyt@mail.kiz.ac.cn

* Correspondence: pcshaw@cuhk.edu.hk

A

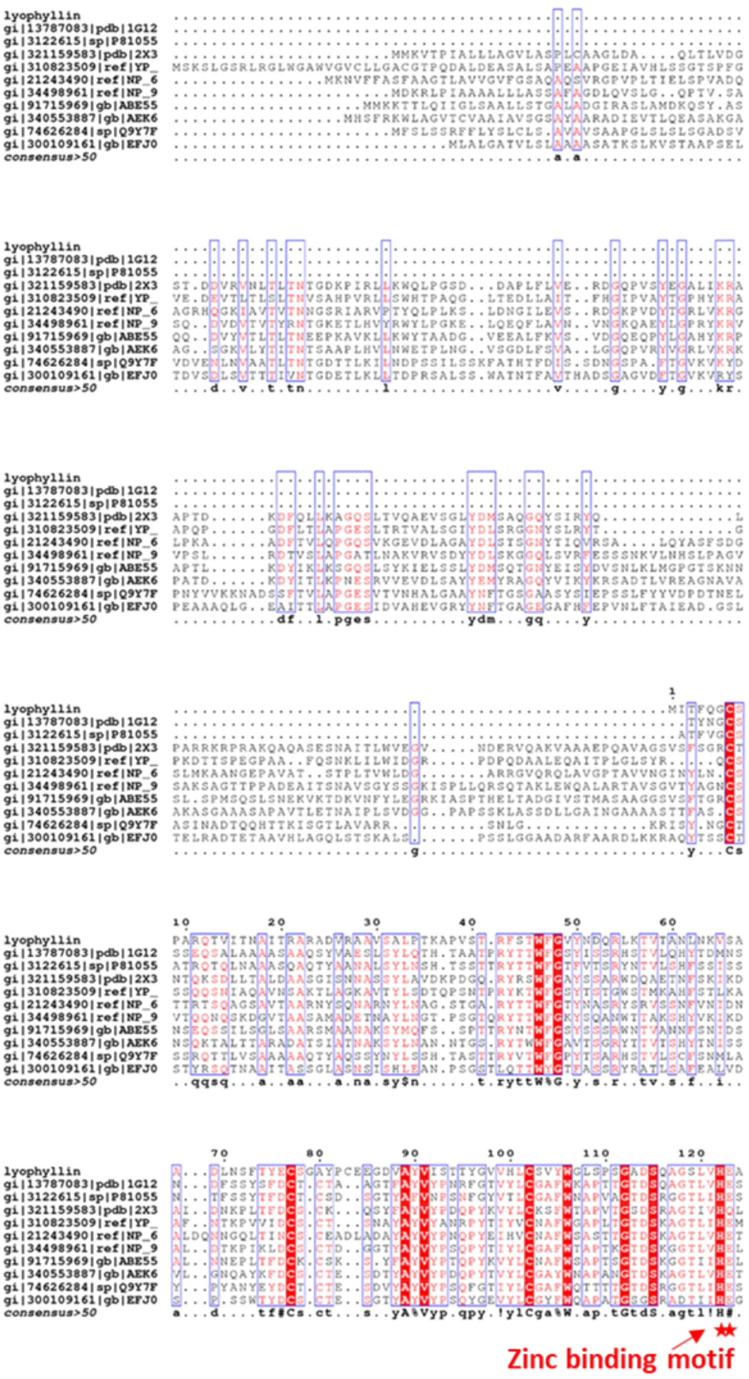

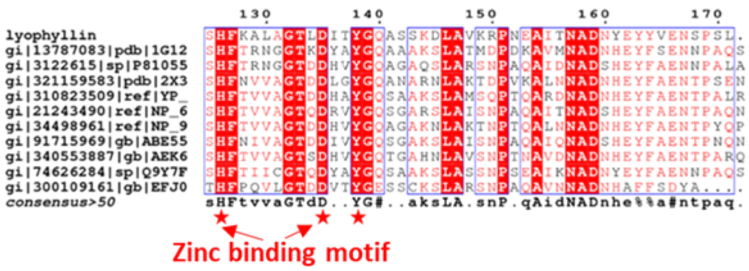

B

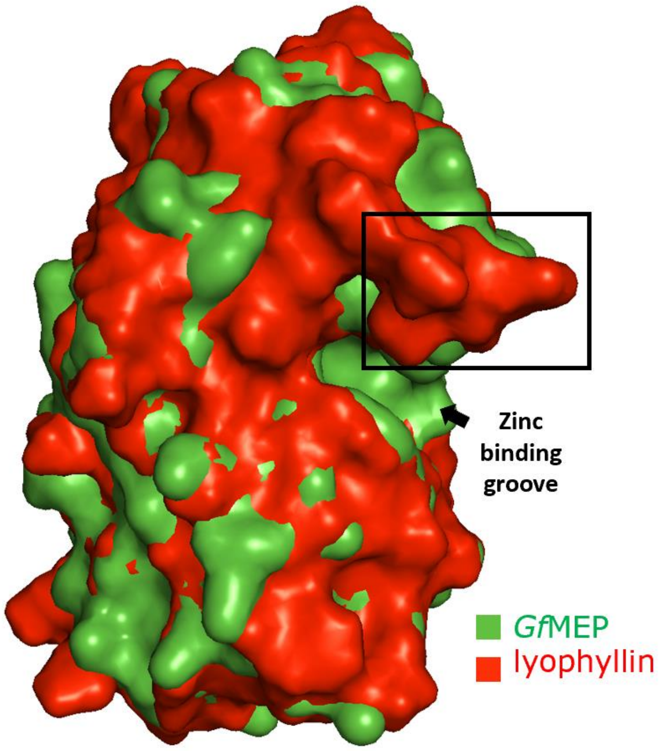

Figure S1. Sequence and structural alignment of lyophyllin with other members from M35 peptidyl-Lys metalloendopeptidases (cd11306: M35_peptidyl-Lys) (A) Sequence of lyophyllin was aligned with other M35 superfamily endopeptidase. The gene/PDB codes were marked in the figure. "HEXXH+D+Y" zinc binding motif is marked in red asterisks. Organisms where the proteins come from are: 1G12_A: *Grifola frondosa*; P81055: *Pleurotus ostreatus*; 2X3A_A: *Aeromonas salmonicida subsp. Achromogenes*; YP_003955867: *Stigmatella aurantiaca*; DW4/3-1; NP_643072: *Xanthomonas axonopodis* pv. citri str. 306; NP_903176: *Chromobacterium violaceum* ATCC 12472; ABE55895: *Shewanella denitrificans* OS217; AEK63262: *Collimonas fungivorans* Ter331; Q9Y7F7: *Armillaria mellea*; EFJ00563: *Schizophyllum commune* H4-8. (B) The superposition of lyophyllin with an GfMEP (PDB code: 1g12), a member of in the M35 endopeptidase superfamily.

Table S1. Primers used in the site-directed mutagenesis study of lyophyllin.

Name	Variant	Forward primer 5′ to 3′	Reverse primer 5′ to 3′
Lyophyllin -wild type	-	TATTATAAGCTTGCATGAT CACTCGTGCGCGCGCCGAC	TTATTACTCGAGTTACAGGG ACGGGGAATT

H121A	H121A	AGCCTGGTTGCTGAAGCTAGT	ACTAGCTTCAGCAACCAGGCT
E122A	E122A	CTGGTTCATGCAGCTAGTCAC	GTGACTAGCTGCATGAACCAG
Y137A	Y137A	GACATTACAGCCGGCCAGGCC	GACATTACAGCCGGCCAGGCC
Y104A	Y104A	TGTTCTGTAGCTTGGGGCCTG	CAGGCCCCAAGCTACAGAACA

A

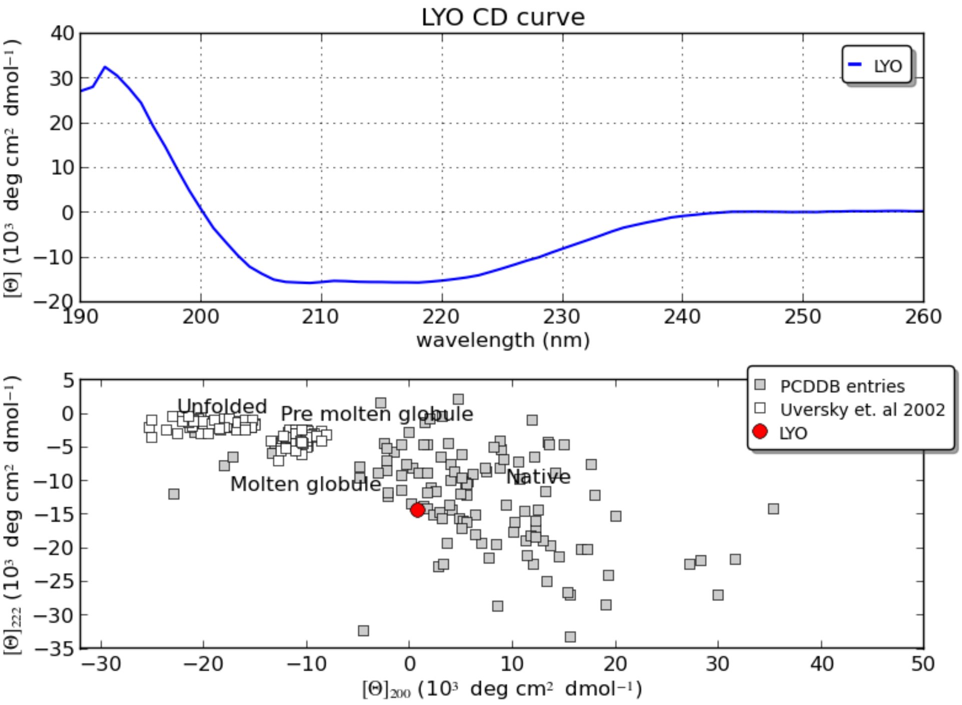

B

	Helix	Strand	Coil
**Experimental data**	0.43	0.06	0.48
**Homology modeling**	0.49	0.09	0.42

Figure S2. Circular dichroism (CD) spectrum and secondary structure comparison of lyophyllin from experimental data and homology modeled structure. (A) CD spectrum of lyophyllin was analyzed by CAPITO (https://data.nmr.uni-jena.de/capito/index.php). (B) Secondary structure of lyophyllin calculated from CD spectrum by CAPITO and the secondary structure from homology modeling are compared.

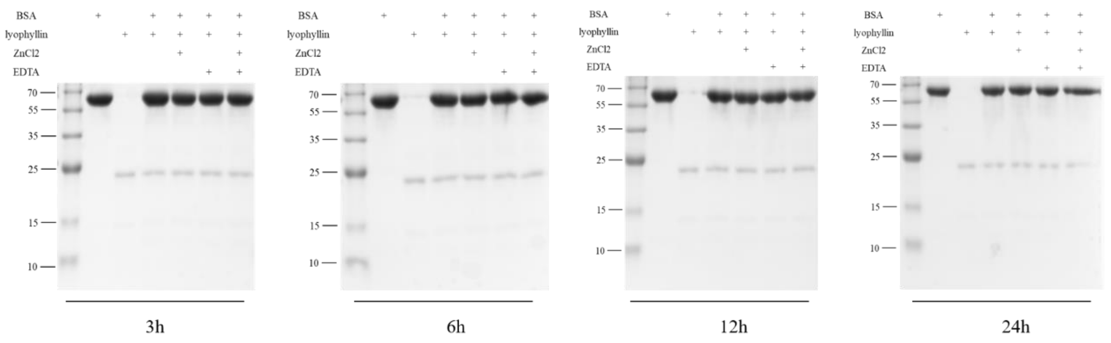

Figure S3. Peptidyl-Lys metalloendopeptidase activity assay of lyophyllin on Bovine serum albumin (BSA). 10 μg of BSA was used in each digestion experiment. The protein samples were digested with lyophyllin at a ratio of 1/50 (w/w) at 25°C for the corresponding time shown in the figure. The reaction mixture was stopped by loading dye and analyzed on 15% SDS-PAGE.

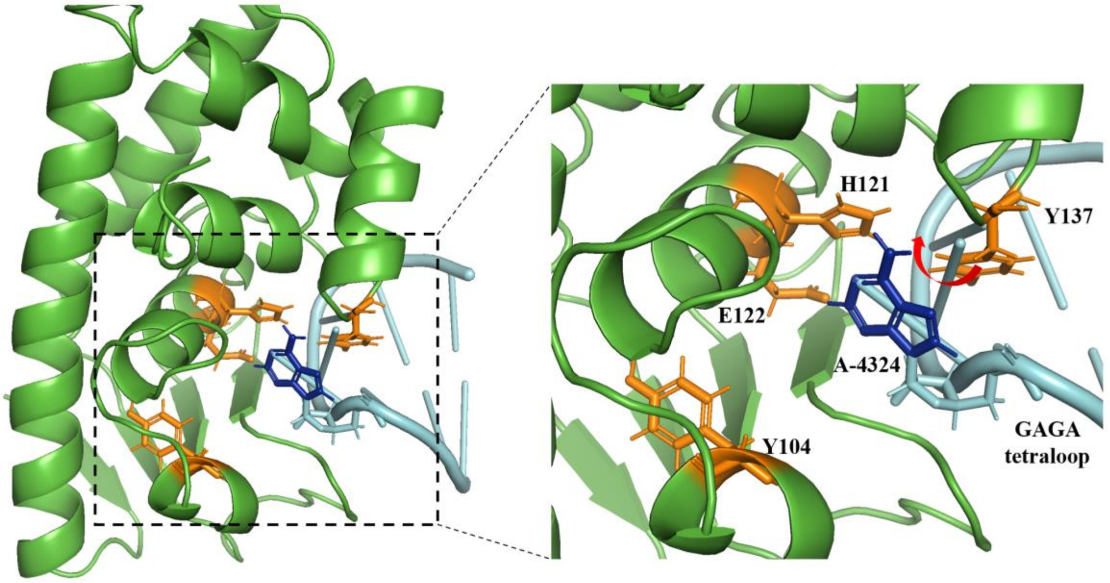

Figure S4. Structural alignment of GAGA hairpin (PDB code: 1ZIG) from α-sarcin/ricin loop to the adenine docked in lyophyllin. GAGA hairpin was structural aligned with the adenine, which was previously docked in lyophyllin by SwissDock as stated in Material and Method. The first A (A-4324) in GAGA may be overlapped with the docked adenine. The little clash can be avoided if Y137 rotates to stack adenosine ring as shown in the red arrow. Lyophyllin is shown in green cartoon, proposed active site amino acid of lyophyllin are shown

in orange sticks. The docked adenine is shown in blue. GAGA hairpin with A-4324 is shown in cyan.
